# An Analysis of EGFR Mutations among 1506 Cases of Non-Small Cell Lung Cancer Patients in Guangxi, China

**DOI:** 10.1371/journal.pone.0168795

**Published:** 2016-12-19

**Authors:** Wen-E Wei, Nai-Quan Mao, Shu-Fang Ning, Ji-Lin Li, Hai-Zhou Liu, Tong Xie, Jian-Hong Zhong, Yan Feng, Chang-Hong Wei, Li-Tu Zhang

**Affiliations:** 1 Department of Research, Affiliated Tumor Hospital of Guangxi Medical University, Nanning, China; 2 Department of Thoracic Surgery, Affiliated Tumor Hospital of Guangxi Medical University, Nanning, China; 3 Department of pathology, Affiliated Tumor Hospital of Guangxi Medical University, Nanning, China; University of Texas MD Anderson Cancer Center, UNITED STATES

## Abstract

An association between epidermal growth factor receptor (EGFR) and clinical characteristics of non-small cell lung cancer (NSCLC) was reported ten years ago. In addition, a different type of relationship was seen in different ethic races. However, the relationship between these factors is not well understood in the Guangxi province. Up to now, there are only very limited data on the association of TTF1/EGFR protein positivity and EGFR mutation status in NSCLC. This study aims to investigate the role of EGFR gene mutation status on the clinical characteristics and the relationship with TTF-1/EGFR protein positivity of patients with NSCLC in Guangxi, China. 1506 samples from different patients with NSCLC were detected by amplification refractory mutation system for 29 hotspot mutations. Analysis of the relationship between clinical characteristics and EGFR mutation status was performed by using the crosstabs Chi-square and SPSS 21.0 software. Of 1506 samples, 537 (35.7%) revealed tyrosine kinase inhibitor (TKI) sensitive EGFR mutations with 27 (1.8%) cases harboring TKI resistant EGFR mutations or union co-existing EGFR-TKIs sensitive mutations. EGFR-TKIs sensitive mutations were not significantly associated with age and TNM-M stage (*P* = 0.863; *P* = 0.572, respectively). However, they were significantly associated with p-stage, TNM-T stage and TNM-N stage (*P* = 0.011, *P <* 0.001, *P* = 0.036, respectively). Immunohistochemical studies revealed that TTF-1 and EGFR protein expression level were all associated with EGFR mutation status (*P* < 0.001, *P* = 0.002, respectively). Of the 537 EGFR-TKIs sensitive mutation cases, the rates of exon 19-del, 18 G719X point, exon 21 L858R and L861Q points were 54.6, 0.9, 42.3 and 0.9%, respectively. EGFR TKI-sensitive mutations commonly occur in female, non-smoking and adenocarcinoma patients. The p-stage, TNM-T stage, TNM-N stage, EGFR and TTF-1 protein expression levels have close relationships with EGFR mutation status.

## Introduction

According to the latest global cancer statistics, about 1.8 million new lung cancer cases occurred in 2012, accounting for nearly 13% of the total cancer diagnoses [1]. Lung cancer was still the leading cause of cancer death among males and the leading cause of cancer among females in more developed countries. It was also the second leading cause of cancer death in less developed countries among females [1]. Non-small cell lung cancers (NSCLCs) account for approximately 85% of primary lung cancer cases [2, 3].

With the emergence of targeted therapy, there are new treatment choices for patients with NSCLCs. Patients with advanced NSCLCs can benefit from the molecular targeted therapeutic drugs such as tyrosine kinase inhibitors (TKIs) [4–6]. The majority of patients who have a good response to TKIs are within a group that possesses the epidermal growth factor receptor (EGFR) active mutation [7–9]. The NSCLC patients with EGFR mutation showed a long progression-free survival (11.5 months) [10] and median survival time (15.4 months) [7] compared to the EGFR mutation negative patients. EGFR mutation was one of the most common mutations in NSCLC patients [11, 12]. The EGFR mutations showed a close relationship with clinical characteristics of patients with lung cancer and mainly appeared in non-smoking female patients with adenocarcinoma lung cancers [12–14]. The frequency of EGFR mutations have a wide variation worldwide, ranging from 31% to 66% in Asian patients [15–17] and 7.5% to 40% in Caucasians [18, 19].

Thyroid transcription factor-1 (TTF-1) is potentially an important marker to distinguish NSCLCs histological subtype. Ninety-five percent of NSCLCs were positive for expression to this marker. In the subtypes of NSCLCs, the expression of TTF-1 in adenocarcinomas is significantly higher than that for squamous cell carcinomas. Previous studies have shown that high TTF-1 expression predicted better prognosis and it can be an independent predictor of favorable prognosis in adenocarcinoma patients [20, 21]. Furthermore, we learnt in previous studies that TTF1-positive tumours are found more frequently in female patients and in non-smokers [22, 23]. It would be interesting to discover whether there is a correlation between TTF1 status and EGFR mutation status in the patients from the Guangxi province of China. Therefore, detection of expression of TTF-1 protein may contribute to the decision whether to use TKIs drugs for subsequent treatment of patients. However, the association between TTF-1 protein expression and EGFR mutation in other ethnicities is not well understood. Therefore, we performed a cross-sectional study with NSCLCs patients and explored the associations between their clinical characteristics and EGFR mutational status.

## Patients and Methods

### Ethics Statements

The local ethics committee of the Affiliated Tumor Hospital of Guangxi Medical University approved this study, and it was conducted in accordance with the Declaration of Helsinki and current ethical guidelines. The patients or the caretakers of patients for their information to be stored in hospital databases and used for research gave written consent. All data underlying the findings described in this manuscript are fully available without restriction.

### Clinical Samples

In this single center study, a total of 1506 NSCLCs lung cancer patients that have taken EGFR mutation tests in exon 18–21 were collected consecutively from August 2010 to June 2016. Those with secondary lung cancer and those patients with a doubtful diagnosis were excluded from the study. All the 1506 samples were collected as fresh resection specimens or paraffin embedding tissue section. Before undergoing EGFR detection, all the patients had not undergone TKIs therapy. All these patients were diagnosed by histodiagnosis. Two board-certified pathologists who examined and verified all the diagnoses crosschecked the results. The criteria used for the pathological diagnosis were set according to the standards of the WHO Classification of Lung Tumors in 2016[24].

In this study, the TNM stage analysis was according to the new lung cancer staging system [25]. At the same time, a p-stage analysis was also performed. Smoking status was classified as non-smokers (<100 lifetime cigarettes), former smokers (quit>1 year prior to diagnosis), or current smokers (still smoking, or quit<1 year prior to diagnosis). EGFR and TTF-1 protein expression were classified as negative and positive.

### EGFR Mutation Detection

EGFR mutations were assessed in all samples within the same laboratory. In this study, all cases were analyzed by amplification refractory mutation system (ARMS) using the human EGFR gene mutations fluorescence polymerase chain reaction diagnostic kit (Amoy Dx, Xiamen, China). This kit is well suited for analyzing specimens such as fresh resection tissue, paraffin embedding tissue sections and peripheral blood. This EGFR kit detects 29 mutations in exons 18 to 21, including T790M, L858R, L861Q, S768I, G719S, G719A, and G719C; three insertions in exon 20; and 19 deletions in exon 19. The kit has its own internal and external controls to monitor the reagents, the quality of DNA obtained and the experimental procedure. In addition, the positive and negative controls would be included along with the samples in each batch of PCR reactions to monitor the cleanliness of environment and correctness of the procedures used. This kit was designed to target the EGFR gene in a relatively conservative section consisting about 100bp, and it even allows for DNA degradation in paraffin embedding tissue sections, such that the internal and external controls can give an estimate of the DNA concentration in the tissue. As usual, the PCR amplification procedure was performed over three stages. The first stage was a 5-minute initial denaturation at 95°C followed by the second stage which consisted of 15 cycles of 25 seconds at 95°C, 20 seconds at 64°C, and 20 seconds at 72°C and the third stage consisted of 31 cycles of 25 seconds at 93°C, 35 seconds at 60°C, and 20 seconds at 72°C.The data were collected at 60°C during stage 3.

DNA from all specimens used were extracted using a TIANamp genomic DNA kit (Tiangen Biotech Co., Ltd, Beijing, China) and all subsequent assays followed the manufacturer’s protocol, and were detected using the Roche LightCycler 480 real-time PCR system. The LightCycle Adapt software (LightCycler 480 Software, v1.5) was use for analysis of the real-time PCR data. The manufacturer’s instructions were used to judge for positive or negative results.

### TTF-1 and EGFR Protein Expression Assessment

The mouse anti-TTF-1 monoclonal antibody kit (ZSGB-BIO, ZM-0270) assessed TTF-1 expression. The mouse anti-EGFR monoclonal antibody kit (ZSGB-BIO, ZM-0093) assessed EGFR protein expression.by. All the operations were in accordance with the manufacturer’s instructions. The TTF-1 protein was expressed positively in neoplastic cell nuclei and this was shown as tan or brown. The EGFR protein was expressed positively in cell membranes and this was shown as pale yellow or brown as a granular uniform distribution. The assessment of TTF-1 and EGFR protein expression levels fell into two categories: TTF-1/ EGFR positive expression and TTF-1/ EGFR negative expression.

### Statistical Analysis

In this study, all the EGFR gene mutation results tested were sorted into two categories: wild type and EGFR mutation positive. In addition, the positive mutations were divided into two sections: EGFR-TKIs sensitive mutations (including exons 18, 19 and 21) and EGFR-TKIs resistant or co-existing mutations (exon 20 or resistant co-existing sensitive mutations). The assessment of TTF-1 and EGFR protein expression levels fell into two categories: TTF-1/ EGFR positive expression and TTF-1/ EGFR negative expression. Analysis of the relationship between clinical characteristics and EGFR mutation status was performed by using the Crosstabs Chi-square and SPSS 21.0 software.

## Results

A total of 1506 patients with NSCLCs were analyzed. The mid-age was 58 years (range from 23–85 years). In all the NSCLCs cases, the patients’ characteristics associated with the mutation status are shown in Table 1.

**Table 1:** Characteristics of patients.

Characteristics	N	EGFR-TKIs SensitiveMutation, (%)	*P* value	EGFR-TKIs resistant or co-existing* Mutation, (%)	*P* value
**Age**					
<65 y	1146	410 (35.8)	0.863	17 (1.5)	0.106
≥65 y	360	127 (35.3)		10(2.8)	
**Gender**					
M	951	268 (28.2)	<0.001	14(1.5)	0.22
F	555	269 (48.5)		13(2.3)	
**Smoking status**					
Never	866	396(45.7)	<0.001	19(2.2)	0.365
Former	121	32 (26.4)		1 (0.8)	
Current	519	109 (21.0)		7(0.8)	
**Histology**					
ADC	1178	493 (41.8)	<0.001	19(1.6)	0.49
ADSC	68	21 (30.9)		3 (4.4)	
SCC	181	12(6.6)		4(2.2)	
Others	79	11 (6.1)		1 (1.3)	
**p-Stage**^1^					
I	192	86 (44.8)	0.011	1(0.5)	0.442
II	133	36 (27.1)		3 (2.3)	
III	343	121 (35.3)		5 (1.5)	
IV	734	272 (37.1)		14 (1.9)	
**TNM**^2^					
**TNM-T**					
T1	276	135 (48.9)	<0.001	0 (0.0)	<0.001
T2	520	207(39.8)		3 (0.6)	
T3	265	69(26.0)		7 (2.6)	
T4	335	101(30.1)		10 (3.0)	
**TNM-N**					
N0	293	110 (37.5)	0.036	6 (2.0)	0.944
N1	127	58 (45.7)		3(2.4)	
N2	545	214 (39.3)		11 (2.0)	
N3	431	141 (32.7)		7(1.6)	
**TNM-M**					
**M0**	686	247(36.0)	0.572	13(1.9)	0.475
**M1**	710	266(37.5)		10(1.4)	
**TTF-1**^3^					
-	242	26 (10.7)	<0.001	2(0.8)	0.698
+	841	373(44.4)		12 (1.4)	
**EGFR protein**^4^					
-	98	26 (26.5)	0.002	2 (2.0)	0.818
+	610	262(43.0)		12(2.0)	

y, year; M, male; F, female; ADC, adenocarcinoma; ADSC, adenosquamous carcinoma; SCC, squamous cell carcinoma;

*, EGFR sensitive mutation coexisting with resistance mutation.

^1^ 104 patients who didn’t provide a p-stage;

^2^ 110 patients who didn’t provide a TNM stage;

^3^ 423 patients missing TTF-1 protein test;

^4^ 798 patients missing the result of the EGFR protein expression.

### Frequency of EGFR Mutations and Clinical Characteristics

The frequency of EGFR mutations associated with clinical characteristics is shown in Table 1. There is no significant difference of EGFR-TKIs sensitive mutations in the age of patients (*p* = 0.863). However, a significant difference was observed with gender (*p* < 0.001). There is no significant difference of EGFR-TKIs resistance or co-exiting mutations in the two genders (*p* = 0.220). In male patients, 28.2% (268/951) harbored EGFR-TKIs sensitive mutations and 1.5% (14/951) harbored resistant or co-existing (sensitive and resistant) mutations. Of 555 female patients, 48.5% (269/555) harbored EGFR-TKIs sensitive mutations and 2.3% (13/555) harbored resistant or co-existing mutations.

There is a significant difference in EGFR-TKIs sensitive mutations when the smoking status of patients was considered (*p* < 0.001). Those who never smoked showed higher mutations than former and current smokers. However, there is no significant difference between former and current smokers (*p* = 0.193). In non-smokers, former smokers and those patients currently smoking, there was 45.7% (396/866), 26.4% (32/121) and21.0% (109/519) patients who harbored EGFR-TKIs sensitive mutations, respectively. The corresponding rates of resistant or co-existing mutations were 2.2% (19/866), 0.8% (1/121) and 0.8% (7/519) respectively. With respect to the resistant or co-existing mutations, there is no statistical difference in different smoking status (*p* = 0.365).

In patients with adenocarcinoma, adenosquamous and squamous-cell carcinoma, 41.8% (493/1178), 30.9% (21/68) and 6.6% (12/181) harbored EGFR-TKIs sensitive mutations (*p* < 0.001), respectively. The corresponding rates of harboring resistant or co-existing mutations were 1.6% (19/1178), 4.4% (3/68) and 2.2% (4/181) harbored resistant or co-existing mutations, respectively (*p* = 0.490).

There were significant differences in the frequency of EGFR-TKIs sensitive mutations in p-stage (*p* = 0.011), TNM-T (*p* < 0.001) and TNM-N (*p* = 0.036). The frequency of EGFR-TKIs sensitive mutations in I, II, III and IV stage were 44.8% (86/192), 27.1% (36/133), 35.3% (121/343) and 37.1% (272/734), respectively. The frequency of EGFR-TKIs sensitive mutations in TNM-T1, TNM-T2, TNM-T3and TNM-T4 stage were 48.9% (135/276), 39.8% (207/520), 26.0% (69/265) and 30.1% (101/335), respectively. The frequency of EGFR-TKIs sensitive mutations in TNM-N0, TNM-N1, TNM-N2, TNM-N3 stage were 37.5% (110/293), 45.7% (58/127), 39.3% (214/545) and 32.7 (141/431), respectively. In additional, there were significant differences in the frequency of EGFR-TKIs resistant or co-existing mutations among TNM-T stage (p < 0.001). The frequency of EGFR-TKIs resistant or co-existing mutations in T1, T2, T3 and T4 were 0% (0/276), 0.6% (3/520), 2.6% (7/265) and 3.0% (10/335), respectively. However, there were no significant differences in the frequency of the EGFR-TKIs resistant or co-existing mutations in p-stage and TNM-N stage (*p* = 0.442, *p* = 0.944, respectively). Furthermore, the frequency of the EGFR mutations showed no significant difference among TNM-M stage (p > 0.05).

### The Relationship between Mutation Status and Immunohistochemistry Analysis

**Fig 1:**
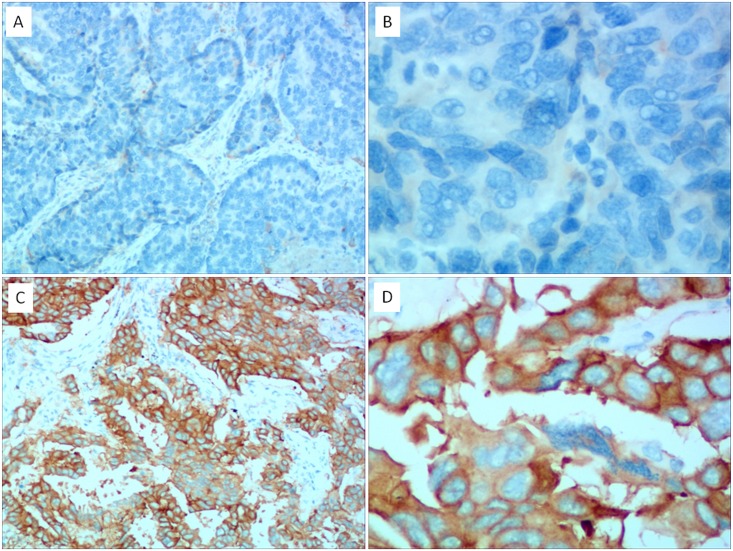
Immunohistochemical staining of TTF-1 expression in non-small cell lung cancer. A: TTF-1 negative (×100); B: TTF-1 negative (×400); C: TTF-1 positive (×100); D: TTF-1 positive (×400).

**Fig 2:**
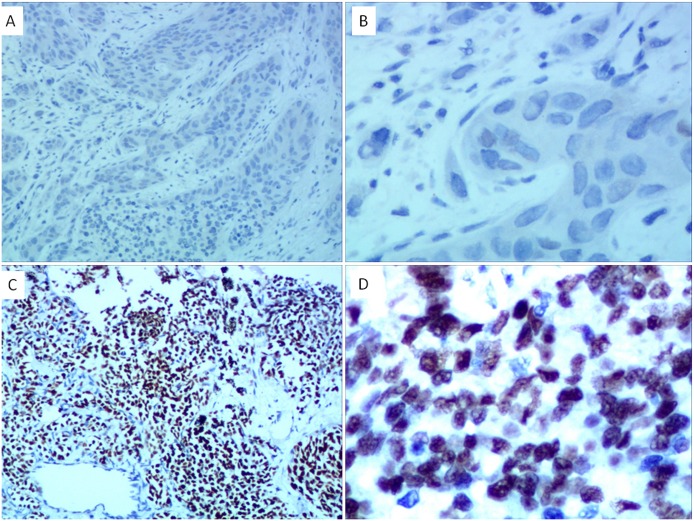
Immunohistochemical staining of EGFR protein expression in non-small cell lung cancer. A: EGFR negative (×100); B: EGFR negative (×400); C: EGFR positive (×100); D: EGFR positive (×400).

TTF-1 and EGFR protein expression were assessed by immunohistochemistry (Figs 1 and 2). There were 423 cases missing TTF-1 and 798 cases missing EGFR protein data. There is a significant difference between TTF-1 protein expression with EGFR mutations (*p* < 0.001). There were 10.7% (26/242) and 44.4% (373/841) in TTF-1 negative and positive patients who harbored EGFR-TKIs sensitive mutations, respectively. In addition, there is a close relationship between EGFR protein expression and EGFR mutation (*p* = 0.002). Only 26.5% (26/98) of EGFR protein negatively expressed samples harbored EGFR sensitive mutations and 43% (262/610) EGFR protein positively expressed samples harbored EGFR sensitive mutations.

### Characteristics of EGFR Mutations

The frequency of EGFR-TKIs sensitive mutations is shown in Table 2. The rate of all samples assessed for EGFR-TKIs sensitive mutations is 35.7% (537/1506) with the most frequent mutation occurring as the exon 19 deletion (19-del) and the exon 21 L858R point mutation (L858R) and the mutation rates were 19.5% (293/1506) and 15.1% (227/1506), respectively. The rates of 19-del and L858R in all cases of EGFR-TKIs sensitive mutation were 54.6% (293/537) and 42.3% (227/537), respectively. With regards to the other types, there were 5 patients who harbored the exon 21 L861Q point mutation (L861Q), 5 patients harbored the exon 18 G719X mutation (G719X) and 7 patients harbored two types of mutation. The mutation rate of 19-del and L858R showed no difference between the genders, smoking status, subtype of histology, TTF-1 and EGFR protein expression (Table 3).

**Table 2:** Frequency of EGFR-TKI sensitive mutations.

Exons	N	% of all study cases (1506)	% of all mutated cases (537)
**Exon 18 (G719X)**	5	0.3	0.9
**Exon 19(19del)**	293	19.5	54.6
**Exon 21(L858R)**	227	15.1	42.3
**Exon 21(L861Q)**	5	0.3	0.9
**Exon 18 and 19del**	2	0.1	0.4
**Exon 18 and L858R**	1	0.07	0.2
**19del/L858R**	4	0.3	0.7
**Total**	537	35.7	100

**Table 3:** The subtype of EGFR mutation 19del/L858R association with clinical characteristics.

Clinic Characteristic	Mutation types		P* *value*
	19-del	L858R	
**Gender**			
**M**	151	112	0.619
**F**	142	115	
**Smoking status**			
**Never**	209	172	0.257
**Former and Current**	84	55	
**Pathology**			
**ADC**	267	213	0.68
**ADSC**	11	7	
**SQC**	8	4	
**TTF-1**			
**-**	12	14	0.367
**+**	199	161	
**EGFR protein**			
**-**	9	16	0.058
**+**	144	114	

M, male; F, female; ADC, adenocarcinoma; ADSC, adenosquamous carcinoma; SCC, squamous cell carcinoma.

*, the difference of prevalence of 19-del/L858R mutation among patients with different characteristics.

The frequency of EGFR-TKIs resistant or co-existing (sensitive and resistant) mutations is shown in Table 4. Approximately 1.8% of samples analyzed harbored EGFR-TKIs resistant or co-existing mutations, and the details are shown in Table 5.

**Table 4:** Frequency of EGFR-TKI resistant or co-exiting* mutations.

Exons	N	(%) Mutation of all study cases (1506)	(%) of all EGFR-TKI resistant and co-exiting* mutation cases (27)
**Exon20 (T790M)**	2	0.1	7.4
**Exon20 (20-ins)**	7	0.5	25.9
**Exon18 and T790M**	1	0.07	3.7
**Exon18/S768I/L861Q**	1	0.07	3.7
**L858R/T790M**	4	0.3	14.8
**L858R/S768I**	1	0.07	3.7
**19del/20-ins**	1	0.07	3.7
**19del/T790M**	3	0.2	11.1
**L858R/L861Q/S768I**	2	0.1	7.4
**S768I/L861Q**	5	0.3	18.5
**Total**	27	1.8	99.9

* EGFR sensitive mutation coexisting with EGFR resistance mutation.

**Table 5:** List of features of each patient whose tumors harbored EGFR TKI resistant mutations.

Subtype	Age (y)	Gender	Smoking status	Tumor Histology
**T790M**	58	M	Never	ADC
	38	F	Never	SCC
**20-ins**	47	M	Never	ADC
	51	M	Current	ADSC
	59	M	Never	ADC
	75	M	Current	ADC
	68	F	Never	ADC
	67	M	Current	ADC
	42	F	Never	ADC
**19-del/20-ins**	66	M	Current	SCC
**19-del/T790M**	73	F	Never	ADC
	73	F	Never	SCC
	64	M	Never	ADC
**L858R/T790M**	51	F	Never	ADC
	43	M	Never	ADC
	50	F	Never	ADC
	72	F	Never	ADC
**L858R/S768I**	55	M	Current	ADC
**G719X/T790M**	46	F	Never	ADC
**S768I/L861Q**	59	M	Current	ADC
	71	M	Former	ADC
	54	M	Never	SCC
	44	F	Never	ADC
	48	F	Never	ADC
**L858R/L861Q/S768I**	61	F	Never	ADSC
	68	M	Current	ADC
**G719X/L861Q/S768I**	67	F	Never	ADC

M, male F; female; ADC, adenocarcinoma; ADSC, adenosquamous carcinoma; SCC, squamous cell carcinoma.

## Discussion

This study shows that the EGFR mutation is common in females, non-smokers and those patients with lung adenocarcinoma. However, no association between EGFR mutation and age was observed. This is in accordance with other studies in this field [15, 16, 19, 26].

In the cohort of this study, we demonstrate the rate of EGFR-TKIs sensitive mutation in NSCLCs is 35.7% (537/1506), a finding which is in accordance with a previous study in China [17]. However, the EGFR-TKIs sensitive mutation rate of 41.8% in lung adenocarcinoma is lower than the rate of 66.3% in a study with a cohort in Southern China [16], but much higher than the mutation rate of 7.5% in the study with a cohort of Northern European populations [27, 28]. The reasons for the difference in different regions may be due to different detection techniques or different populations having different gene backgrounds.

Gender and smoking status both show significant differences with mutation status, respectively. According to other studies [18, 19, 27], it is clear that the high rate of EGFR mutation in females is similar to the high rate of EGFR mutation in the non-smoking population. The EGFR mutation rate in males resembles the rate in the smoking population. Therefore, we have reasons to believe that the different genders have different distributions depending on their smoking status (smokers are mainly distributed in males) and this is one reason that accounts for the difference in mutation rates among the two genders. Other reasons maybe the variations of the physiological makeup of males and females. However, the mechanisms by which gender and smoking status contribute to EGFR mutation is still largely unknown. In addition, the interactions between gender and smoking status still remain unknown.

With regards to the EGFR mutations in the different histological subtypes, this study showed that the highest EGFR-TKIs sensitive mutation frequency occurred in adenocarcinoma (41.8% (493/1178)) and this was followed by adenosquamous carcinoma, (30.9% (21/68)), squamous cell carcinoma (6.6% (12/181)) and others (6.1% (11/79)). There is a significant difference with respect to EGFR-TKIs sensitivity in the various subtypes. However, there is no significant difference regarding EGFR-TKIs resistant or co-existing mutations in the various subtypes. It is clear that the occurrence of EGFR-TKIs sensitive mutation in non-adenocarcinoma was relatively rare. In addition, a previous study reported that the patients who expressed sensitive mutations in non-adenocarcinoma responded poorly and non-effectively to the EGFR-TKIs [29], and hence whether it is even a necessity to undergo a routine mutation test in the absence of adenocarcinomas. However, in the study of Chiu and coworkers, they pointed out that it is useful to have an EGFR mutation test for those patients who have a poor performance status or other comorbidities [30]. In this study, although the rate of EGFR-TKIs sensitive mutation in squamous cell carcinoma and adenosquamous carcinoma was obviously lower than with adenocarcinoma, but it is clear that the rate is higher than other geographical regions [27]. Therefore, we would advise that there is a necessity to have an EGFR mutation test no matter what subtypes are presented, especially in those patients who failed to get benefit from other therapies but where EGFR-TKIs target therapy is being administered.

With respect to the patients’ database, no significant difference between the mutation occurrence frequency and age and TNM-M stage were found, a result which is similar to those found in other studies [13, 15, 26]. Interestingly, in this study, we find that there are remarkable significant differences between TNM-T stage and EGFR mutations (EGFR-TKIs sensitive and EGFR-TKIs resistant or co-existing ones) (*p* < 0.001; *p* < 0.001). In addition, there are marked significant differences between the EGFR-TKIs sensitive mutations occurrence frequency and p-stage, TNM-N stage (*p* = 0.011, *p* = 0.036). The EGFR-TKIs sensitive mutation occurred mainly in TNM-T1 stage and the EGFR-TKIs sensitive or co-existing mutations occurred mainly in TNM-T4 stage, respectively. The rate that EGFR-TKIs sensitive mutations in T1, T2, T3 and T4 form an obvious trend of gradient descent from 48.9% (135/276), 39.8% (207/520), 26.0% (69/265) to 30.1% (101/335), respectively. The rate that EGFR-TKIs resistant or co-existing mutations in T1, T2, T3 and T4 form an obvious trend of rising gradient from 0.0% (0/276), 0.6% (3/520),2.6% (7/265) to 3.0% (10/335), respectively. However, the rate of EGFR-TKIs sensitive mutations in I-IV stage and N0-N3 stage show no obvious trend of change.

Based on our analysis, we can hypothesis that the patients with a smaller *in situ* tumor diameter can be more suited for surgery without any other medical treatment and therefore can avoid the effect of medicines on gene detection. In addition, in the patients with a larger *in situ* tumor diameter and hence are at an advance stage of lung cancer, are most likely to be inoperable. Those patients with a large tumor diameter may well have had chemo- or radiotherapy to shrink the tumor diameter to suitable size for surgery. Therefore, we can see that the patients with smaller diameter tumors show a high rate of EGFR-TKIs sensitive mutation and the patients with larger diameter tumors show a high rate of EGFR-TKIs resistant or co-existing mutations. Therefore, it is important to detect an EGFR mutation as early as possible, and this will determine the formulation of treatment for the individual patient.

There is some controversy regarding the relationship between EGFR protein expression and EGFR gene mutation. A study in a cohort of 119 Norwegian patients showed that there is no significant relationship between EGFR protein expression and EGFR gene mutation [27]. However, the study in a cohort of Korean showed a close relationship between EGFR protein expression and EGFR gene mutation [31]. In the present study, 1506 cases were examined which is relatively larger than the above studies, and EGFR protein expression level showed a close relationship with EGFR-TKIs sensitive mutation with EGFR protein positive-expression being more frequent in the patients who harbored EGFR mutation. The size of sample cases studied maybe one reason for the different results seen. Other reasons that may account for the differences are ethnic groups or differences in experimental methods and evaluation systems employed. It is probable that gene mutations can lead to high gene expression levels and this would in turn cause EGFR protein positive-expression. Therefore, it is probable that the EGFR protein expression is closely related to EGFR-TKIs sensitive mutation.

TTF-1 is a nucleoprotein with a molecular weight of 40kD and is one of the members of the gene transcription NKx2 family. It was discovered as a transcriptional regulator of thyroglobulin in 1989, and the first studies in relation to lung cancer were conducted in 2007 [32].

Previous studies have shown that the patients with positive TTF-1 protein expression can show improvement in lung adenocarcinoma survival [33–35]. In this study, we find that TTF-1 protein expression show a close relationship with EGFR mutation. Positive TTF-1 expression was more frequent in those patients who harbored EGFR mutation. This result is in accordance with studies performed in Chinese, Korean and Caucasian populations [18, 31, 36]. It is not always possible to detect EGFR gene mutation when the tissue samples obtained are in a poor condition and in those cases it is useful to evaluate TTF-1 or EGFR protein expression as this may give a valid basis for the development of strategies for clinical care for patients with NSCLC.

With respect to the EGFR mutation subgroup analysis, we can demonstrate that 19-del and L858R are the two main subtypes of EGFR-TKIs sensitive mutations in Chinese patients with NSCLCs, including 54.6% and 42.3% of all the EGFR-TKIs sensitive mutations, respectively. There is no significant difference between 19-del and L858R (*p* = 0.296).This result is in accordance with the study in a cohort of the Chinese population by Lai and coworkers[17]. However, this result is inconsistent with other studies that investigated a cohort of Caucasian[18, 37, 38]. In the mutation cases, exon 21 and 19 mutation rates show no significant difference with gender, smoking status, pathology, TTF-1 and EGFR protein expression. This result is not consistent with the Japanese study where it was reported that exon 19 deletion mainly occurs in male and exon 21 mainly occurs in females [37].

Although this manuscript has analyzed the status of EGFR gene mutations and EGFR/TTF-1 protein expression levels on 1506 cases of NSCLC patients, the lack of prognoses data and biochemical mechanism research limited the scope of this study.

## Conclusions

In summary, in this study based on a large cohort of patients, there were three interesting findings. Firstly, the p-stage has a close relationship with EGFR-TKIs sensitive mutations with the patients who in I stage have the most frequency EGFR-TKIs sensitive mutations. Secondly, the TNM-T stage has a close relationship with EGFR mutations, and that the early TNM-T stage shows more common EGFR-TKIs sensitive mutations whereas the late TNM-T stage shows more common EGFR-TKIs resistant or co-existing mutations. Thirdly, the TNM-N stage has a close relationship with EGFR-TKIs sensitive mutations even though the rate among the N0-N3 show no remarkable changing trend. With respect to the result of the IHC, the TTF-1 and EGFR protein levels showed a close relationship with EGFR-TKIs sensitive mutations. In addition, TTF-1 and EGFR protein positive-expression was associated with an increased frequency of mutations.

## Supporting Information

S1 File The original data of this study. (XLSX)

## References

[1] Torre LA, Bray F, Siegel RL, Ferlay J, Lortet-Tieulent J, Jemal A. Global cancer statistics, 2012. CA: a cancer journal for clinicians. 2015;65(2):87–108. 25651787 10.3322/caac.21262

[2] He YY, Zhang XC, Yang JJ, Niu FY, Zeng Z, Yan HH, et al Prognostic Significance of Genotype and Number of Metastatic Sites in Advanced Non-Small-Cell Lung Cancer. Clin Lung Cancer. 2014;15(6):441–7. 10.1016/j.cllc.2014.06.006 25044104

[3] Chen Z, Fillmore CM, Hammerman PS, Kim CF, Wong KK. Non-small-cell lung cancers: a heterogeneous set of diseases. Nat Rev Cancer. 2014;14(8):535–46. 10.1038/nrc3775 25056707 PMC5712844

[4] Kuiper JL, Heideman DA, Wurdinger T, Grunberg K, Groen HJ, Smit EF. Rationale and study design of the IRENE-trial (NVALT-16): a phase II trial to evaluate iressa rechallenge in advanced NSCLC patients with an activating EGFR mutation who responded to an EGFR-TKI used as first-line or previous treatment. Clinical lung cancer. 2015;16(1):60–6. 10.1016/j.cllc.2014.07.008 25242669

[5] Lee JK, Hahn S, Kim DW, Suh KJ, Keam B, Kim TM, et al Epidermal growth factor receptor tyrosine kinase inhibitors vs conventional chemotherapy in non-small cell lung cancer harboring wild-type epidermal growth factor receptor: a meta-analysis. Jama. 2014;311(14):1430–7. 10.1001/jama.2014.3314 24715074

[6] Sunaga N, Tomizawa Y, Yanagitani N, Iijima H, Kaira K, Shimizu K, et al Phase II prospective study of the efficacy of gefitinib for the treatment of stage III/IV non-small cell lung cancer with EGFR mutations, irrespective of previous chemotherapy. Lung Cancer. 2007;56(3):383–9. 10.1016/j.lungcan.2007.01.025 17368623

[7] Sutani A, Nagai Y, Udagawa K, Uchida Y, Koyama N, Murayama Y, et al Gefitinib for non-small-cell lung cancer patients with epidermal growth factor receptor gene mutations screened by peptide nucleic acid-locked nucleic acid PCR clamp. British journal of cancer. 2006;95(11):1483–9. 10.1038/sj.bjc.6603466 17106442 PMC2360739

[8] Asahina H, Yamazaki K, Kinoshita I, Sukoh N, Harada M, Yokouchi H, et al A phase II trial of gefitinib as first-line therapy for advanced non-small cell lung cancer with epidermal growth factor receptor mutations. British journal of cancer. 2006;95(8):998–1004. 10.1038/sj.bjc.6603393 17047648 PMC2360715

[9] Ramalingam SS, Blackhall F, Krzakowski M, Barrios CH, Park K, Bover I, et al Randomized phase II study of dacomitinib (PF-00299804), an irreversible pan-human epidermal growth factor receptor inhibitor, versus erlotinib in patients with advanced non-small-cell lung cancer. Journal of clinical oncology: official journal of the American Society of Clinical Oncology. 2012;30(27):3337–44. 22753918 10.1200/JCO.2011.40.9433 PMC5321098

[10] Tamura K, Okamoto I, Kashii T, Negoro S, Hirashima T, Kudoh S, et al Multicentre prospective phase II trial of gefitinib for advanced non-small cell lung cancer with epidermal growth factor receptor mutations: results of the West Japan Thoracic Oncology Group trial (WJTOG0403). British journal of cancer. 2008;98(5):907–14. 10.1038/sj.bjc.6604249 18283321 PMC2266849

[11] Lynch TJ, Bell DW, Sordella R, Gurubhagavatula S, Okimoto RA, Brannigan BW, et al Activating mutations in the epidermal growth factor receptor underlying responsiveness of non-small-cell lung cancer to gefitinib. The New England journal of medicine. 2004;350(21):2129–39. 10.1056/NEJMoa040938 15118073

[12] Paez JG, Janne PA, Lee JC, Tracy S, Greulich H, Gabriel S, et al EGFR mutations in lung cancer: correlation with clinical response to gefitinib therapy. Science. 2004;304(5676):1497–500. 10.1126/science.1099314 15118125

[13] Kosaka T, Yatabe Y, Endoh H, Kuwano H, Takahashi T, Mitsudomi T. Mutations of the epidermal growth factor receptor gene in lung cancer: biological and clinical implications. Cancer Res. 2004;64(24):8919–23. 10.1158/0008-5472.CAN-04-2818 15604253

[14] Mitsudomi T, Kosaka T, Yatabe Y. Biological and clinical implications of EGFR mutations in lung cancer. International journal of clinical oncology. 2006;11(3):190–8. 10.1007/s10147-006-0583-4 16850125

[15] Tanaka T, Matsuoka M, Sutani A, Gemma A, Maemondo M, Inoue A, et al Frequency of and variables associated with the EGFR mutation and its subtypes. International journal of cancer Journal international du cancer. 2010;126(3):651–5. 10.1002/ijc.24746 19609951

[16] Gao B, Sun Y, Zhang J, Ren Y, Fang R, Han X, et al Spectrum of LKB1, EGFR, and KRAS mutations in chinese lung adenocarcinomas. Journal of thoracic oncology: official publication of the International Association for the Study of Lung Cancer. 2010;5(8):1130–5. 10.1097/JTO.0b013e3181e05016 PMC4009449 20559149

[17] Lai Y, Zhang Z, Li J, Sun D, Zhou Y, Jiang T, et al EGFR mutations in surgically resected fresh specimens from 697 consecutive Chinese patients with non-small cell lung cancer and their relationships with clinical features. International journal of molecular sciences. 2013;14(12):24549–59. 10.3390/ijms141224549 24351833 PMC3876127

[18] Gahr S, Stoehr R, Geissinger E, Ficker JH, Brueckl WM, Gschwendtner A, et al EGFR mutational status in a large series of Caucasian European NSCLC patients: data from daily practice. British journal of cancer. 2013;109(7):1821–8. 10.1038/bjc.2013.511 24002608 PMC3790166

[19] Dogan S, Shen R, Ang DC, Johnson ML, D'Angelo SP, Paik PK, et al Molecular epidemiology of EGFR and KRAS mutations in 3,026 lung adenocarcinomas: higher susceptibility of women to smoking-related KRAS-mutant cancers. Clinical cancer research: an official journal of the American Association for Cancer Research. 2012;18(22):6169–77. 23014527 10.1158/1078-0432.CCR-11-3265 PMC3500422

[20] Xu XY, Yang GY, Yang JH, Li J. Analysis of clinical characteristics and differential diagnosis of the lung biopsy specimens in 99 adenocarcinoma cases and 111 squamous cell carcinoma cases: utility of an immunohistochemical panel containing CK5/6, CK34betaE12, p63, CK7 and TTF-1. Pathology, research and practice. 2014;210(10):680–5. 10.1016/j.prp.2014.06.021 25063315

[21] Perner S, Wagner PL, Soltermann A, LaFargue C, Tischler V, Weir BA, et al TTF1 expression in non-small cell lung carcinoma: association with TTF1 gene amplification and improved survival. The Journal of pathology. 2009;217(1):65–72. 10.1002/path.2443 18932182

[22] Anagnostou VK, Syrigos KN, Bepler G, Homer RJ, Rimm DL. Thyroid Transcription Factor 1 Is an Independent Prognostic Factor for Patients With Stage I Lung Adenocarcinoma. J Clin Oncol. 2009;27(2):271–8. 10.1200/JCO.2008.17.0043 19064983

[23] Sun PL, Seol H, Lee HJ, Yoo SB, Kim H, Xu X, et al High Incidence of EGFR Mutations in Korean Men Smokers with No Intratumoral Heterogeneity of Lung Adenocarcinomas Correlation with Histologic Subtypes, EGFR/TTF-1 Expressions, and Clinical Features. J Thorac Oncol. 2012;7(2):323–30. 10.1097/JTO.0b013e3182381515 22237264

[24] Micke P, Mattsson JSM, Djureinovic D, Nodin B, Jirstrom K, Tran L, et al The Impact of the Fourth Edition of the WHO Classification of Lung Tumours on Histological Classification of Resected Pulmonary NSCCs. J Thorac Oncol. 2016;11(6):862–72. 10.1016/j.jtho.2016.01.020 26872818

[25] Detterbeck FC, Boffa DJ, Tanoue LT. The new lung cancer staging system. Chest. 2009;136(1):260–71. 10.1378/chest.08-0978 19584208

[26] Helland A, Skaug HM, Kleinberg L, Iversen ML, Rud AK, Fleischer T, et al EGFR Gene Alterations in a Norwegian Cohort of Lung Cancer Patients Selected for Surgery. J Thorac Oncol. 2011;6(5):947–50. 10.1097/JTO.0b013e31820db209 21623266

[27] Helland A, Skaug HM, Kleinberg L, Iversen ML, Rud AK, Fleischer T, et al EGFR gene alterations in a Norwegian cohort of lung cancer patients selected for surgery. Journal of thoracic oncology: official publication of the International Association for the Study of Lung Cancer. 2011;6(5):947–50. 10.1097/JTO.0b013e31820db209 21623266

[28] Hegi ME, Diserens AC, Bady P, Kamoshima Y, Kouwenhoven MC, Delorenzi M, et al Pathway analysis of glioblastoma tissue after preoperative treatment with the EGFR tyrosine kinase inhibitor gefitinib—a phase II trial. Molecular cancer therapeutics. 2011;10(6):1102–12. 10.1158/1535-7163.MCT-11-0048 21471286

[29] Shek D, Longmate J, Quinn DI, Margolin KA, Twardowski P, Gandara DR, et al A phase II trial of gefitinib and pegylated IFNalpha in previously treated renal cell carcinoma. International journal of clinical oncology. 2011;16(5):494–9. 10.1007/s10147-011-0212-8 21431345

[30] Chiu CH, Chou TY, Chiang CL, Tsai CM. Should EGFR mutations be tested in advanced lung squamous cell carcinomas to guide frontline treatment? Cancer chemotherapy and pharmacology. 2014;74(4):661–5. 10.1007/s00280-014-2536-3 25053390

[31] Sun PL, Seol H, Lee HJ, Yoo SB, Kim H, Xu X, et al High incidence of EGFR mutations in Korean men smokers with no intratumoral heterogeneity of lung adenocarcinomas: correlation with histologic subtypes, EGFR/TTF-1 expressions, and clinical features. Journal of thoracic oncology: official publication of the International Association for the Study of Lung Cancer. 2012;7(2):323–30. 10.1097/JTO.0b013e3182381515 22237264

[32] Mu D. The complexity of thyroid transcription factor 1 with both pro- and anti-oncogenic activities. The Journal of biological chemistry. 2013;288(35):24992–5000. 10.1074/jbc.R113.491647 23818522 PMC3757165

[33] Barletta JA, Perner S, Iafrate AJ, Yeap BY, Weir BA, Johnson LA, et al Clinical significance of TTF-1 protein expression and TTF-1 gene amplification in lung adenocarcinoma. Journal of cellular and molecular medicine. 2009;13(8B):1977–86. 10.1111/j.1582-4934.2008.00594.x 19040416 PMC2830395

[34] Barlesi F, Pinot D, Legoffic A, Doddoli C, Chetaille B, Torre JP, et al Positive thyroid transcription factor 1 staining strongly correlates with survival of patients with adenocarcinoma of the lung. British journal of cancer. 2005;93(4):450–2. 10.1038/sj.bjc.6602717 16052216 PMC2361585

[35] Boccardo F, Rubagotti A, Conti G, Battaglia M, Cruciani G, Manganelli A, et al Prednisone plus gefitinib versus prednisone plus placebo in the treatment of hormone-refractory prostate cancer: a randomized phase II trial. Oncology. 2008;74(3–4):223–8. 10.1159/000151391 18714171

[36] Shanzhi W, Yiping H, Ling H, Jianming Z, Qiang L. The relationship between TTF-1 expression and EGFR mutations in lung adenocarcinomas. PloS one. 2014;9(4):e95479 10.1371/journal.pone.0095479 24743427 PMC3990660

[37] Tokumo M, Toyooka S, Kiura K, Shigematsu H, Tomii K, Aoe M, et al The relationship between epidermal growth factor receptor mutations and clinicopathologic features in non-small cell lung cancers. Clinical cancer research: an official journal of the American Association for Cancer Research. 2005;11(3):1167–73. 15709185

[38] Sonobe M, Manabe T, Wada H, Tanaka F. Mutations in the epidermal growth factor receptor gene are linked to smoking-independent, lung adenocarcinoma. British journal of cancer. 2005;93(3):355–63. 10.1038/sj.bjc.6602707 16052218 PMC2361570

